# Introducing *Journal of Biological Engineering*

**DOI:** 10.1186/1754-1611-1-1

**Published:** 2007-10-10

**Authors:** Mark R Riley

**Affiliations:** 1 Department of Agricultural and Biosystems Engineering, The University of Arizona, Tucson, AZ 85721, USA

## 

Welcome to *Journal of Biological Engineering (JBE)*, the flagship publication of the Institute of Biological Engineering.

Biological Engineering is a new and unique discipline that encompasses a wide range of engineering theory and practice connected to and derived from biology. This emerging science-based engineering discipline and related applications are experiencing rapid growth. Prior to publication of *JBE *there is no refereed publication that encompasses the breadth and depth of biological engineering as a science-based discipline, rather than a collection of applications. Our goal in publishing *JBE *is to provide a forum for topics that address the foundational questions that unify all applications of biological engineering.

In this editorial, we aim to describe the field of biological engineering and how it continues to evolve into a unique discipline sprouting from a common core. The role and scope of *JBE *will also be discussed.

## What is biological engineering and how does it differ from other fields?

Biological engineering is a science-based engineering discipline that requires a strong grounding in biology as well as in goal based analysis and design. Engineering fields typically are rooted to one fundamental science. The difficulty in defining biological engineering on this basis is that the field is not merely applied biology since information flows in both directions from the basic science to applied engineering and back again. Biological Engineering (BE) is not just biology influenced by engineering design or engineering approaches applied to biological systems [1]; it is more than that.

The integration of biology into engineering and engineering into biology [2] has not been as clear a path as that taken previously in the development of applied chemistry and physics due in part to biology's uncertain rules and inherent variability. BE can be differentiated from its roots of pure biology or classical engineering in the following way. Biological studies often follow a reductionist approach in viewing a system on its smallest possible scale which naturally leads toward tools such as functional genomics. Engineering approaches, using classical design perspectives, are constructionist, building new devices, approaches, and technologies from component concepts. Biological engineering utilizes **both **of these methods in concert relying on reductionist approaches to define the fundamental units which are then commingled in non-natural ways to generate something new.

A distinction must be made between biological science and biological engineering. Several definitions are relevant here.

"Biological engineering is the optimization of the performance of a task or set of tasks performed by a biological system through the application of the engineering design process," [2].

An academic department at MIT presents that the goal for the "biological engineering discipline is to advance fundamental understanding of how biological systems operate and to develop effective biology-based technologies for applications across a wide spectrum of societal needs including breakthroughs in diagnosis, treatment, and prevention of disease, in design of novel materials, devices, and processes, and in enhancing environmental health," [3].

The NIH definition of the related area of bioengineering was presented on July 24, 1997. "Bioengineering integrates physical, chemical, or mathematical sciences and engineering principles for the study of biology, medicine, behavior, or health. It advances fundamental concepts, creates knowledge for the molecular to the organ systems levels, and develops innovative biologics, materials, processes, implants, devices, and informatics approaches for the prevention, diagnosis, and treatment of disease, for patient rehabilitation, and for improving health," [4].

The NIH definition of bioengineering is focused on human health, which is a critical component of biological engineering. But the broader discipline of biological engineering also addresses the full spectrum of the life sciences, including applications to agricultural, environmental, and ecological systems that utilize engineering approaches and rationale to solve biological problems [5]. Industrial applications of BE stretch beyond medicine to transform biological feedstocks through value-added processes into ethanol, hydrogen, biodiesel, nutraceuticals, bioplastics and others.

Analogies can be drawn between biological engineering design and the technologies and approaches developed in other engineering disciplines, often based on the principles of standardization, decoupling, and abstraction [6]. Microorganisms can now be "programmed" using interchangeable constructs of DNA that confer logic and operational commands, enabling the creation of synthetic biological devices and systems with new functionality [7]. The microbes retain many of their native characteristics including the abilities to reproduce, respond to stimuli, and repair minor damage. The implications of such hybrid organisms are enormous as machines can be designed and built with the capabilities to evolve – with all the associated positive and negative implications. Some of the challenges faced by synthetic biology parallel many of the difficulties faced by environmental restoration and ecological engineering, in that the ultimate embodiment of a design may differ significantly from that conceived by the designer. Nature tends to follow its own path to a new steady state.

BE is unique from other disciplines because of such emergent behavior – biology is more than applied chemistry. A living organism or ecosystem is more than the sum of its parts. A phase shift appears when one moves in length scale from sub-cellular to multi-cellular while analysis of shorter time scales reveals complexity as population behavior deconstructs into molecular probabilities. Practioners of BE needs to examine networks and multi-level connections first amongst molecules, then across structural and temporal motifs, then finally to create new functionalities that integrate synergistic properties.

A key component of BE is that it enables new approaches to manufacturing. The breadth of relevant manufacturing processes ranges from synthesizing new organisms or developing a new bioconversion process to restoring a wetland. Processes are not independent. Heat does not merely flow out of a control volume, but it then enters an external environment which cannot be ignored or assumed to play a passive role. Biologically engineered processes, since they utilize living systems, have the necessity of being performed using sustainable methods. They must utilize renewable resources for materials and energy, wastes must be reused and recycled, and end use states must be designed to leave no negative footprint. A process that is fully engineered while taking account of the biology must not only address economics and market pressures, but also incorporate carbon emissions, waste utilization, and the impact of these activities on the surrounding ecosystem addressed from a systems perspective [7].

One of the reasons for launching *JBE *at this time is that the field is sufficiently mature that higher level discussions can be made amongst practioners to define the foundational core concepts, express the current breadth of the field, and to connect developments from disparate application areas which share a fundamental foundation. An appropriate approach to foster the development of biological engineering is to identify underlying phenomena that govern the behavior of biological and biologically derived systems regardless of scale, environment, or application.

## Role and scope of JBE

*JBE *manuscripts will integrate engineering with life sciences to generate new quantitative methods, models, and information. Peer reviewed articles will encompass cutting edge research that fully integrates fundamental inquiry, quantitative analysis, and translational design. *JBE *provides a home for the continuum from biological information science, molecules and cells, organismal studies, product formation, wastes and remediation, and education. A description of the broader context of the work and how it connects to other areas must be incorporated into each manuscript.

Topical areas include (but are not be limited to):

• Synthetic biology and cellular design

• Engineering of devices that interface at the biomolecular, cellular, and organismal levels

• Bioproduction and bioproduct engineering,

• Ecological and environmental engineering, and

• Biological engineering education and the biodesign process.

*JBE *invites manuscript submissions that address theoretical and applied approaches to design, optimize, and use biological systems ranging in scale from molecules, cells, organisms, to ecosystems. *JBE *will incorporate advances in mathematical sciences (including computational and statistical methods) enabling the development of biological engineering, biological engineering design inspired by or in the context of biology, and designs for all scales that include nanometer to ecosystem levels.

We have lofty goals for *JBE*; we aim for this to become the preferred home for cutting edge research in biological engineering. To provide a platform to unify the field it is necessary that all manuscripts in *JBE *not only be of high quality, but that they also present a discussion of how the work advances biological engineering, drives new development and application, or connects disparate areas in new ways. This can be accomplished in many ways and likely will evolve in time.

*JBE *also will be a home for educational advances in curriculum content and pedagogy at the undergraduate and graduate-levels.

The publisher, BioMed Central, is an open access [8] provider, where the authors retain copyright of their manuscript. There are no subscription fees or fees to access articles once published; instead articles are freely available immediately upon publication. To offset the cost of publication authors pay an article-processing charge (APC) [9]. Authors who do not have sufficient funds to pay the APC may request a discretionary full or partial waiver from BioMed Central.

To foster and nurture the development of biological engineering, we have launched this publication as an open access, online resource. Open access is an important tenet of biological engineering in that information not only is to be freely accessible to the scientific community, but it must be available to the public at large.

Established in 1995, the Institute of Biological Engineering is an independent society, which aims to encourage inquiry, application, and interest in biological engineering in the broadest and most liberal manner, and to promote the professional development of its members. The society promotes the development of engineering science in the context of biology that adds to the fundamental knowledge and principles ubiquitous in all designs and application domains. Hallmarks of IBE conferences include offering a unique forum for broadly based discussions on biological engineering and facilitating communications related to biology inspired engineering design.
